# The AMPK-related kinase SIK2 is regulated by cAMP via phosphorylation at Ser^358^ in adipocytes

**DOI:** 10.1042/BJ20111932

**Published:** 2012-05-29

**Authors:** Emma Henriksson, Helena A. Jones, Kashyap Patel, Mark Peggie, Nicholas Morrice, Kei Sakamoto, Olga Göransson

**Affiliations:** *The Department of Experimental Medical Science, Lund University, BMC C11, 221 84 Lund, Sweden; †MRC Protein Phosphorylation Unit, College of Life Sciences, University of Dundee, Dow Street, Dundee DD1 5EH, Scotland, U.K.; ‡The Beatson Institute for Cancer Research, Garscube Estate, Switchback Road, Bearsden, Glasgow G61 1BD, Scotland, U.K.

**Keywords:** 14-3-3, cAMP, insulin, phosphorylation, salt-induced kinase (SIK), 3T3-L1 adipocyte, AICAR, 5-amino-4-imidazolecarboxamide riboside, AMPK, AMP-activated protein kinase, CaMK, Ca^2+^/calmodulin-dependent kinase, COX, cytochrome *c* oxidase, CREB, cAMP-response-element-binding protein, CRTC2, CREB-regulated transcription co-activator-2, DMEM, Dulbecco's modified Eagle's medium, DSTT, Division of Signal Transduction Therapy, DTT, dithiothreitol, ERK, extracellular-signal-regulated kinase, FBS, fetal bovine serum, GAPDH, glyceraldehyde-3-phosphate dehydrogenase, GFP, green fluorescent protein, GST, glutathione transferase, HA, haemagglutinin, HDAC, histone deacetylase, HEK, human embryonic kidney, HRP, horseradish peroxidase, HSL, hormone-sensitive lipase, IBMX, isobutylmethylxanthine, IP, immunoprecipitate, IRS1, insulin receptor substrate 1, LC, liquid chromatography, MAPK, mitogen-activated protein kinase, MS/MS, tandem MS, NLS, nuclear localization signal, PK, protein kinase, SIK, salt-inducible kinase, TBS-T, Tris-buffered saline containing 0.2% Tween 20

## Abstract

SIK2 (salt-inducible kinase 2) is a member of the AMPK (AMP-activated protein kinase) family of kinases and is highly expressed in adipocytes. We investigated the regulation of SIK2 in adipocytes in response to cellular stimuli with relevance for adipocyte function and/or AMPK signalling. None of the treatments, including insulin, cAMP inducers or AICAR (5-amino-4-imidazolecarboxamide riboside), affected SIK2 activity towards peptide or protein substrates *in vitro*. However, stimulation with the cAMP-elevating agent forskolin and the β-adrenergic receptor agonist CL 316,243 resulted in a PKA (protein kinase A)-dependent phosphorylation and 14-3-3 binding of SIK2. Phosphopeptide mapping of SIK2 revealed several sites phosphorylated in response to cAMP induction, including Ser^358^. Site-directed mutagenesis demonstrated that phosphorylation of Ser^358^, but not the previously reported PKA site Ser^587^, was required for 14-3-3 binding. Immunocytochemistry illustrated that the localization of exogenously expressed SIK2 in HEK (human embryonic kidney)-293 cells was exclusively cytosolic and remained unchanged after cAMP elevation. Fractionation of adipocytes, however, revealed a significant increase of wild-type, but not Ser358Ala, HA (haemagglutinin)–SIK2 in the cytosol and a concomitant decrease in a particulate fraction after CL 316,243 treatment. This supports a phosphorylation-dependent relocalization in adipocytes. We hypothesize that regulation of SIK2 by cAMP could play a role for the critical effects of this second messenger on lipid metabolism in adipocytes.

## INTRODUCTION

SIK (salt-inducible kinase) 2, also called QIK (Qin-induced kinase), is an AMPK (AMP-activated protein kinase)-related kinase family member [1] with abundant expression in adipocytes [2,3]. SIK2, and the third isoform SIK3 (also known as QSK), was identified by homology with SIK1 [3,4]. Although the highest expression of SIK2 is found in adipose tissue [2,3], SIK1 is abundant in adrenal glands [5–7] and SIK3 displays a more ubiquitous expression pattern [3].

An important step in elucidating the biological function of SIK2 and its related kinases is to determine how they are affected by different cellular signals. The most well-characterized mode of regulation for SIK2 so far discovered is the phosphorylation of its activation (T-) loop Thr^175^ by LKB1, which is required for SIK2 catalytic activity, as demonstrated by the complete lack of activity in the Thr175Ala mutant, or in LKB1-deficient cells [8]. The T-loop phosphorylation site of SIK1 and SIK3, but not SIK2, binds to 14-3-3 scaffolding proteins, which in turn regulates the activity and localization of these kinases [9]. Agents that increase the AMP/ATP ratio have been shown not to influence SIK2 activity in various systems [8,10]. On the other hand, one study reported that AICAR (5-amino-4-imidazolecarboxamide riboside), an AMP mimetic, as well as glucose starvation, stimulated SIK2 activity in 3T3-L1 adipocytes [2]; however, the underlying mechanism for this activation was not described. Another report proposes that insulin activates SIK2 in hepatocytes via phosphorylation of Ser^358^ by PK (protein kinase) B, and that this regulation in part mediates the ability of insulin to inhibit gluconeogenesis [11]. More recently, SIK2 was suggested to be regulated by CaMK (Ca^2+^/calmodulin-dependent kinase) I/IV in neuronal cells, via a phosphorylation of Thr^484^ [12]. The cAMP/PKA pathway has been shown to regulate SIK isoforms, in particular SIK1. Treatment of Y1 cells and 3T3-L1 fibroblasts with cAMP-elevating agents was demonstrated to induce the phosphorylation of SIK1 on Ser^577^ (human Ser^575^), resulting in its nuclear export [7,13]. The homologous site of SIK2, Ser^587^, was similarly phosphorylated; however, the effect on SIK2 localization was not as clear as for SIK1, probably owing to the lack of an NLS (nuclear localization signal) in SIK2 [3,14]. The phosphorylation of SIK1 and 2 in response to cAMP induction is thought to restrict their inhibitory action on different transcriptional regulators, including CREB (cAMP-response-element-binding protein) co-activator CRTC2 (CREB-regulated transcription co-activator-2) [previously called TORC2 (transducer of regulated CREB activity 2)] [14,15].

As mentioned above, the expression of SIK2 is many-fold higher in adipose tissue than elsewhere, and is induced during adipocyte differentiation [3]. In addition, SIK2 protein expression and activity were also shown to be up-regulated in adipose tissue of *db*/*db* mice. This, along with its described role in other tissues and relationship to AMPK, a known target of anti-diabetic drugs, prompted us to carefully investigate the regulation of SIK2 in adipocytes. Various cellular signals with important roles in the regulation of AMPK and/or adipocyte function were studied and we present evidence that cAMP, a critical second messenger in the control of lipid metabolism, regulates SIK2 in adipocytes at many levels.

## EXPERIMENTAL

### Materials

3T3-L1 cells were from A.T.C.C. and DMEM (Dulbecco's modified Eagle's medium), FBS (fetal bovine serum), dexamethasone, IBMX (isobutylmethylxanthine), insulin (differentiation of 3T3-L1 fibroblasts), phenformin, forskolin, ionomycin, CL 316,243, tetracycline, fish skin gelatine and HA (haemagglutinin)–agarose were all from Sigma. Flp-in™ T-REx™-293 cells, Flp-recombinase, blasticidin B, hygromycin, Hoechst nuclear stain, pre-cast Novex SDS/PAGE Bis-Tris gels and SDS sample buffer were all from Invitrogen. Penicillin/streptomycin was from VWR. Complete protease inhibitor cocktail was from Roche and AICAR was from Toronto Research Chemicals. H89 was from Biomol and insulin (for stimulation) was from Novo Nordisk. Protein G–Sepharose and glutathione–Sepharose were from GE Healthcare. [γ-^32^P]ATP was from PerkinElmer and phosphocellulose P81 paper was from Whatman. Hydromount was from National Diagnostics. AMARA peptide, HDAC5tide (where HDAC is histone deacetylase) and IRS1tide (where IRS1 is insulin receptor substrate1) were synthesized by Dr Grahame Bloomberg (University of Bristol, Bristol, U.K.) and Sakamototide was synthesized by Pepceuticals. Adenoviral vectors for HA–SIK2 wild-type, Ser358Ala and Ser587Ala were produced by Vector BioLabs.

### Antibodies

The following primary antibodies were used for Western blotting: anti-[HSL (hormone-sensitive lipase) phosphoSer^563^], anti-AMPK, anti-(AMPK phosphoThr^172^), anti-COX (cytochrome *c* oxidase) IV, anti-phosphoPKA substrate and anti-phosphoPKB substrate antibodies were all from Cell Signalling Technology. Anti-(PKB phosphoSer^473^) was from Invitrogen, anti-HA, anti-GAPDH (glyceraldehyde-3-phosphate dehydrogenase) and anti-FLAG–HRP (horseradish peroxidase) antibodies were all from Sigma, anti-14-3-3 antibody was from Santa Cruz Biotechnology and anti-H3 antibody was from Millipore. Anti-(Na/K ATPase) antibody was from Novus Biologicals. The following antibodies were raised in rabbit and affinity-purified by Innovagen: anti-SIK2 (residues 906–926 of human SIK2, LFDCEMLDAVDPQHNGYVLVN) used for immunoprecipitation and immunoblotting and anti-(SIK2 phosphoSer^358^) (residues 351–365 of human SIK2, DGRQRRPpSTIAEQTV) used for immunoblotting. An antibody against phosphorylated T-loop SIK isoforms (residues 175–189 of human SIK1) was raised in sheep, and purified by the DSTT (Division of Signal Transduction Therapy), University of Dundee, Dundee, U.K. The anti-BMH1/2 antibody used for 14-3-3 far-Western bloting was a gift from Professor Carol MacKintosh, University of Dundee, Dundee, U.K. Anti-AMPKα1 antibody for the immunoprecipitation kinase assay was provided by Professor D. Grahame Hardie, University of Dundee, Dundee, U.K. Secondary antibodies conjugated with HRP were from Invitrogen (anti-rabbit), Pierce (anti-sheep) and GE Healthcare (anti-mouse). Alexa Fluor™ 594 anti-mouse antibody was purchased from Invitrogen.

### cDNA constructs

pGEX expression constructs encoding human HDAC5 (1–550) and 14-3-3ζ, cDNA constructs for human wild-type, kinase inactive (Lys49Met), Thr175Ala HA–SIK2, wild-type, Ser358Ala GFP (green fluorescent protein)–SIK2 and Ser575Ala HA– and GFP–SIK1, as well as the N- and C-terminally tagged wild-type and Ser358Ala SIK2–FLAG pcDNA5/FRT/TO constructs were generated by the DSTT. HDAC5 (GenBank® accession number NM_005474.4) was amplified from IMAGE clone 6043491 (Source Bioscience) using KOD Hot Start DNA polymerase (Novagen), cloned into pSC-b (Stratagene) and sequenced. This was then digested with BglII/Not1 and cloned into the BamH1/Not1 sites of pGEX6P-1. Mutants were created using the QuikChange® method (Stratagene), but using the KOD Hot Start DNA polymerase. SIK constructs were as described previously [8,9]. Ser587Ala GFP–SIK2 was subcloned from dCCM Ser587Ala HA–SIK2. The following dCCM (Vector Biolabs) expression constructs encoding phosphorylation site mutant forms of human HA–SIK2 were generated by DNA Cloning Service: Ser343Ala, Ser358Ala, Thr484Ala and Ser587Ala. dCCM wild-type HA–SIK2 was subcloned from pCMV5 wild-type HA–SIK2.

### Culture, differentiation and stimulation of 3T3-L1 adipocytes

3T3-L1 fibroblasts were cultured and differentiated as described previously [16]. After stimulation, the cells were rinsed with PBS and harvested in ice-cold lysis buffer containing 50 mM Tris/HCl (pH 7.5), 1 mM EGTA, 1 mM EDTA, 1% (w/v) Nonidet P40, 1 mM sodium orthovanadate, 10 mM sodium-2-glycerophosphate, 50 mM sodium fluoride, 5 mM sodium pyrophosphate, 0.27 M sucrose, 1 mM DTT (dithiothreitol) and complete protease inhibitor (one tablet/50 ml). Following centrifugation at 4°C for 15 min at 13000 ***g***, infranatants were collected and total protein content was determined using the Bradford assay.

### Isolation, transduction and stimulation of primary rat adipocytes

Adipocytes were prepared from epididymal adipose tissue as described previously [16], under a protocol approved by the ethical review committee at Lund University (number M212-09). Adenoviral expression of wild-type, Ser358Ala or Ser587Ala HA–SIK2 [50×10^6^ pfu (plaque-forming units)/ml, 100 MOI (multiplicity of infection)] was performed overnight in DMEM containing 5% FBS, 1% penicillin/streptomycin and 0.5% BSA at 37°C. Adipocytes were subsequently resuspended in KRH (Krebs–Ringer–Hepes), defined in [16], containing 1% BSA and stimulated in a 37°C shaking water bath. Lysates were prepared as described previously [16].

### Subcellular fractionation of primary rat adipocytes

Primary rat adipocytes expressing wild-type or Ser358Ala HA–SIK2 were isolated, stimulated and homogenized in ice-cold homogenization buffer containing 30 mM Tris/HCl (pH 7.5), 1 mM EGTA, 1 mM EDTA, 0.25 mM sodium orthovanadate, 10 mM sodium-2-glycerophosphate, 10 mM sodium fluoride, 5 mM sodium pyrophosphate, 0.27 M sucrose, 1 mM DTT and complete protease inhibitor. The homogenates were centrifuged at 80 ***g*** for 5 min at 4°C to remove the cell debris and triacylglycerols and then further centrifuged at 60000 ***g*** for 45 min at 4°C. The supernatant (referred to as ‘cytosol’) was supplemented with 1% Nonidet P40, after which insoluble particles were removed by centrifugation at 13000 ***g*** for 15 min at 4°C. The resulting pellet (60000 ***g***, referred to as the ‘particulate fraction’) was resuspended in homogenization buffer containing a final concentration of 1% Nonidet P40, after which the insoluble particles were removed by centrifugation as described above.

### Generation of stable cell lines

Flp-in™ T-REx™-293 cells cultured in 10-cm dishes were transfected with 0.5 μg of pcDNA5/FRT/TO-FLAG constructs encoding N- or C-terminally FLAG-tagged wild-type or Ser358Ala SIK2 and 4.5 μg of cDNA encoding Flp recombinase. After 3 days, DMEM selection medium containing 1% penicillin/streptavidin, 10% FBS, 15 μg/ml blasticidin B and 100 μg/ml hygromycin was added. The medium was changed every 24 h until only cells expressing wild-type or Ser358Ala FLAG–SIK2 remained. Selection medium was kept throughout experiments. Cells were stimulated and lysed (described below) 12 h after induction with 0.1 μg/ml tetracycline.

### Culture, transfection and stimulation of HEK (human embryonic kidney)-293 cells

HEK-293 cells cultured in 10-cm dishes were transfected with 2 μg of cDNA construct encoding wild-type or the indicated mutant forms of HA– or GST (glutathione transferase)–SIK2, using the polyethylenimine method [17]. At 24 h post-transfection, the cells were starved for 16 h, stimulated, rinsed twice with PBS and lysed in 0.5 ml of ice-cold lysis buffer described above. Lysates were centrifuged at 13000 ***g*** for 15 min to remove cell debris. GST fusion proteins were affinity-purified on glutathione–Sepharose and eluted in 50 mM Tris/HCl (pH 7.5), 0.1 mM EGTA, 1 mM DTT, 0.27 M sucrose and 20 mM glutathione.

### Measurement of SIK2 kinase activity

Cell lysates (5–50 μg) were incubated for 1 h on a shaking platform with 3 μg of anti-SIK2 or 1 μg of anti-AMPKα1 antibody conjugated to 5 μl of packed Protein G–Sepharose, alternatively anti-HA–agarose. The IPs (immunoprecipitates) were then further processed as described in [16]. Phosphotransferase activity of immunoprecipitated or purified recombinant (10 ng) SIK2 (and AMPK) towards the peptides AMARA (AMARAASAAALARRR), a commonly used peptide substrate for measuring AMPK activity based on the AMPK consensus peptide substrate sequence [18], Sakamototide [19] (ALNRTSSDSALHRRR, residues 165–179, R177,178,179 of human CRTC2), which encompasses Ser^171^ of CRTC2, known to be phosphorylated by AMPK and SIK isoforms [15], HDAC5tide (PLRKTASEPNLKRRR, residues 253–267, R265,266,267 of human HDAC5) and IRS1tide (HLRLSTSSGRLLRRR, residues 788–802, R800,801,802 of human IRS1), which encompass Ser^259^ and Ser^794^ respectively, in these proteins [3,20,21], was then measured as described previously [16]. The stoichiometry of HDAC5tide phosphorylation in the GST–SIK2 assay was ~0.2, and the *K*_m_ value for ATP was ~90 μM. Using 100 μM, a maximum of 6% of the ATP was consumed. Subsequently, 200 μM HDAC5tide and Sakamototide was used for measuring SIK2 kinase activity in the cell lysates. Phosphotransferase activity of SIK2 towards the protein substrate HDAC5 (1–550) was measured in a total assay volume of 25 μl containing 50 mM Tris/HCl (pH 7.5), 0.1% 2-mercaptoethanol, 10 mM MgCl_2_, 0.1 mM EGTA, 0.1 mM [γ-^32^P]ATP (300 c.p.m./pmol) and 0.5 μg of HDAC5 (1–550) protein for 20 min at 30°C. Following polyacrylamide gel electrophoresis and colloidal Coomassie Blue staining, the gels were fixed, dried and analysed in an FLA 3000 Fujifilm reader using Fuji imaging plates type BAS-III. One unit of activity was defined as that which catalysed the transfer of 1 nmol of ^32^P/min to the substrate.

### Western and far-Western blot analysis

Cell lysates or IPs prepared from 50–500 μg of total protein, as described above, or pull downs prepared by the addition of 5 μg of *Escherichia coli*-expressed GST–14-3-3ζ bound to glutathione–Sepharose (described below), were heated in SDS sample buffer, before loading on to a precast 4–12% Novex gradient gel and transferred on to a nitrocellulose membrane as described previously [16]. Detection was performed using HRP-conjugated secondary antibodies and ECL (enhanced chemiluminescence) reagent. Far-Western membranes were incubated overnight with 5 μg/ml recombinant yeast His–14-3-3 (BMH1/2) produced in *E. coli*, in TBS-T (Tris-buffered saline containing 0.2% Tween 20) containing 5% (w/v) BSA and 0.5 M NaCl and then washed in TBS-T containing 0.5 M NaCl. An anti-His antibody was used to detect His–14-3-3. Films were developed and quantified as described previously [16].

### Expression and purification of HDAC5 (1–550) and 14-3-3ζ in *E. coli*

pGEX expression constructs encoding human-GST-fused HDAC5 (1–550) and 14-3-3ζ were transformed into *E. coli* BL21 cells and expressed and purified as described previously [9] with minor modifications. The IPTG (isopropyl β-D-thiogalactopyranoside) concentration used was 250 μM.

### Phosphopeptide mapping of SIK2 and identification of 14-3-3 isoforms by mass fingerprinting

Wild-type and mutant forms of HA–SIK2 were expressed in HEK-293 cells and primary rat adipocytes as described above. SIK2 immunoprecipitated from 10 mg of total protein was loaded on to a polyacrylamide gel, which was subsequently stained with colloidal Coomassie Blue. The bands representing HA–SIK2 and 14-3-3 were excised, incubated with 50 mM iodoacetamide to alkylate cysteine residues, washed and digested with trypsin for 16 h. Tryptic digests were analysed by LC (liquid chromatography)–MS with precursor of 79 scanning on an Applied Biosystems 4000 Q-Trap system as described in [22]. SIK2 tryptic digests were also analysed by LC–MS on either an LTQ-orbitrap classic mass spectrometer system (HEK-293 cells) or an LTQ-orbitrap Velos mass spectrometer system (adipocytes) each coupled to a Proxeon Easy-LC HPLC system. The peptide mixtures were separated on either an LC-Packings PepMap C_18_ column (0.075 mm×150 mm) (HEK-293 cells) or a Proxeon EASY-Column (0.075 mm×100 mm) (adipocytes) equilibrated in 0.1% formic acid/water and eluted with a discontinuous gradient of acetonitrile/0.1% formic acid at a flow rate of 300 nl/min. The orbitrap was set to analyse the survey scans at 60000 resolution and top 5 ions in each duty cycle, were selected for MS/MS (tandem MS) in the LTQ linear ion trap with multistage activation.

The MS/MS spectra were searched against the SwissProt database using the Mascot search engine (Matrix Science) run on an in-house server using the following criteria: peptide tolerance=10 p.p.m., trypsin as the enzyme, and carboxyamidomethylation of cysteine as a fixed modification with oxidation of methionine and phosphorylation of serine, threonine and tyrosine as a variable modification. Any MS/MS spectra that could be assigned to a phosphopeptide were inspected manually using QualBrowser software.

### Confocal microscopy of HEK-293 cells expressing HA- or GFP-tagged SIK1 and SIK2

HEK-293 cells were cultured on cover slips in six-well plates and transfected as described above using 0.5 μg of cDNA. At 16 h post-transfection, cells were stimulated, washed with PBS and fixed using 4% formaldehyde. For the HA constructs, the cells were permeabilized using PBS containing 0.25% Triton X-100 and then blocked in fish skin gelatin (3%) for 30 min before primary antibody was added. Secondary antibody (Alexa Fluor™ 594) was added after three washes with PBS containing 0.1% Tween. Hoechst nuclear stain was added in one of the final washes before mounting the cover slips on slides using Hydromount. For confocal imaging, a Zeiss LSM 510 META microscope was used with excitation wavelengths 405 (Hoechst nuclear stain), 488 (GFP) or 561 (Alexa Fluro™ 594) nm. A Plan-Apochromat 63×/1.4 oil DIC (differential interference contrast) objective was used and a frame size of 488 pixels×488 pixels×1 pixels.

### Statistical methods and calculation of *V*_max_ and *K*_m_ values

Results are means±S.D. and analyses were performed using GraphPad Prism 5. A non-linear regression using a Michaelis–Menten model was used for determining *V*_max_ and *K*_m_ values and includes S.E.M. The statistic significance of differences was defined as *P<0.05, **P<0.01 and ***P<0.001 and determined using a paired two-tailed Student's *t* test.

## RESULTS

### Increased cAMP levels in adipocytes results in a PKA-dependent phosphorylation of SIK2 with no effect on its intrinsic kinase activity

**Figure 1:**
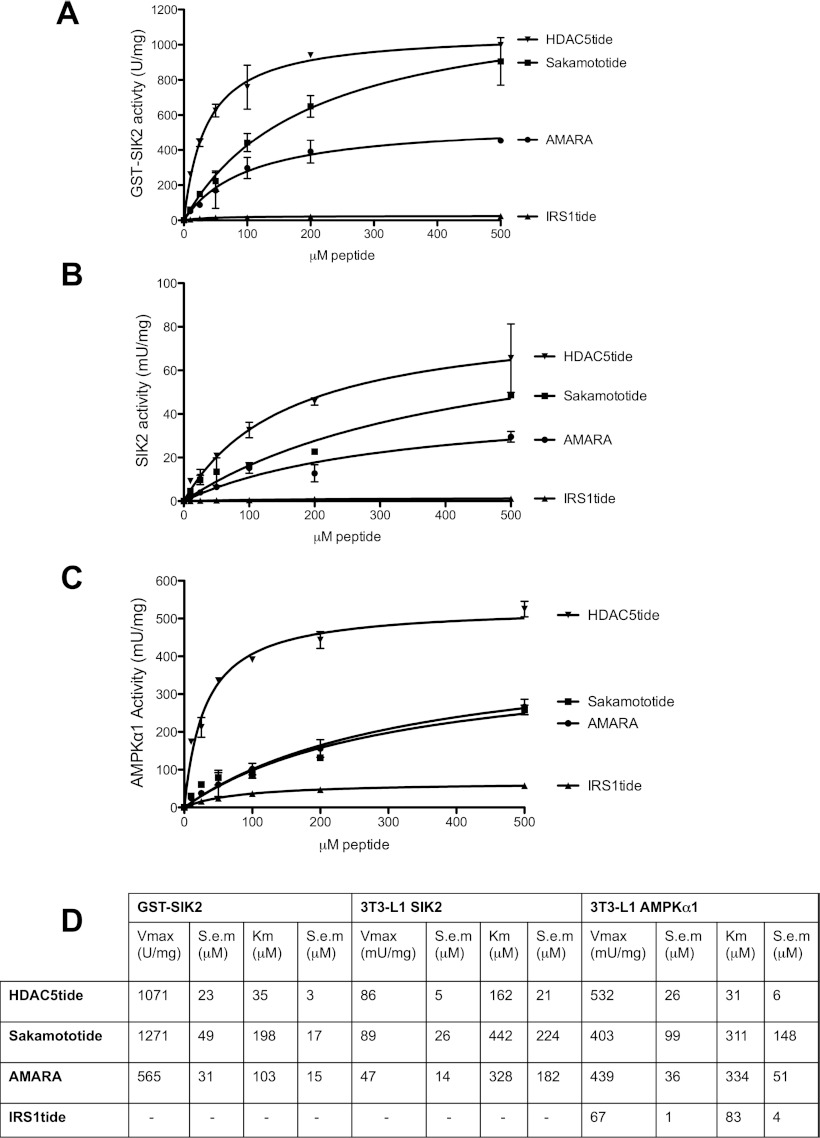
Identification of suitable peptide substrates for *in vitro* kinase activity measurements of SIK2 (**A**) Purified wild-type or kinase-inactive (Lys49Met, not shown) GST–SIK2 (10 ng) expressed in HEK-293 cells was assayed *in vitro* using the following substrate concentrations: 0, 10, 25, 50, 100, 200 and 500 μM. (**B** and **C**) Endogenous SIK2 or AMPKα1 immunoprecipitated from 3T3-L1 adipocytes was used to evaluate the peptide substrates under the same conditions as for GST–SIK2. Results are means±S.D. for a triplicate assay. (**D**) Non-linear regression using a Michaelis–Menten model was used for determining *V*_max_ and *K*_m_ values using GraphPad Prism 5.

The kinase domain of SIK isoforms and AMPK are well conserved and SIK has been shown to phosphorylate AMPK-targeted sequences in both intact cells and in cell-free assays. In order to identify more selective and efficient substrates for the measurement of SIK2 activity, we evaluated four peptides (described further in the Experimental section) derived from known AMPK- and SIK-targeted residues in ACC (acetyl-CoA carboxylase; AMARA), CRTC2 (Sakamototide), HDAC5 (HDAC5tide) and IRS1 (IRS1tide). All substrates were tested using purified recombinant wild-type or kinase-inactive (Lys49Met, results not shown) GST–SIK2 expressed in HEK-293 cells (Figure 1A), as well as endogenous SIK2 immunoprecipitated from 3T3-L1 adipocyte cell extracts (Figure 1B). Endogenous AMPKα1 from 3T3-L1 adipocytes was used as a reference (Figure 1C). HDAC5tide was identified as the most efficient peptide substrate for measurements of both recombinant and endogenous SIK2 kinase activity on the basis of its high *V*_max_ (GST–SIK2, 1071 units/mg and 3T3-L1 adipocyte SIK2, 86 m units/mg) and low *K*_m_ values (GST–SIK2, 35 μM and 3T3-L1 adipocyte SIK2, 162 μM) (Figures 1A, 1B and 1D), whereas both recombinant and endogenous SIK2 failed to display phosphotransferase activity towards IRS1tide. For AMPKα_1_, HDAC5tide was also shown to be an optimal substrate (Figures 1C and 1D). We confirmed that recombinant SIK2 phosphorylates a single site on Sakamototide (Ser^171^) and HDAC5tide (Ser^259^), using [γ-^32^P]ATP-labelled peptides followed by analysis with Edman sequencing (results not shown). HDAC5tide and Sakamototide were selected as peptide substrates for SIK2 kinase activity measurements.

**Figure 2:**
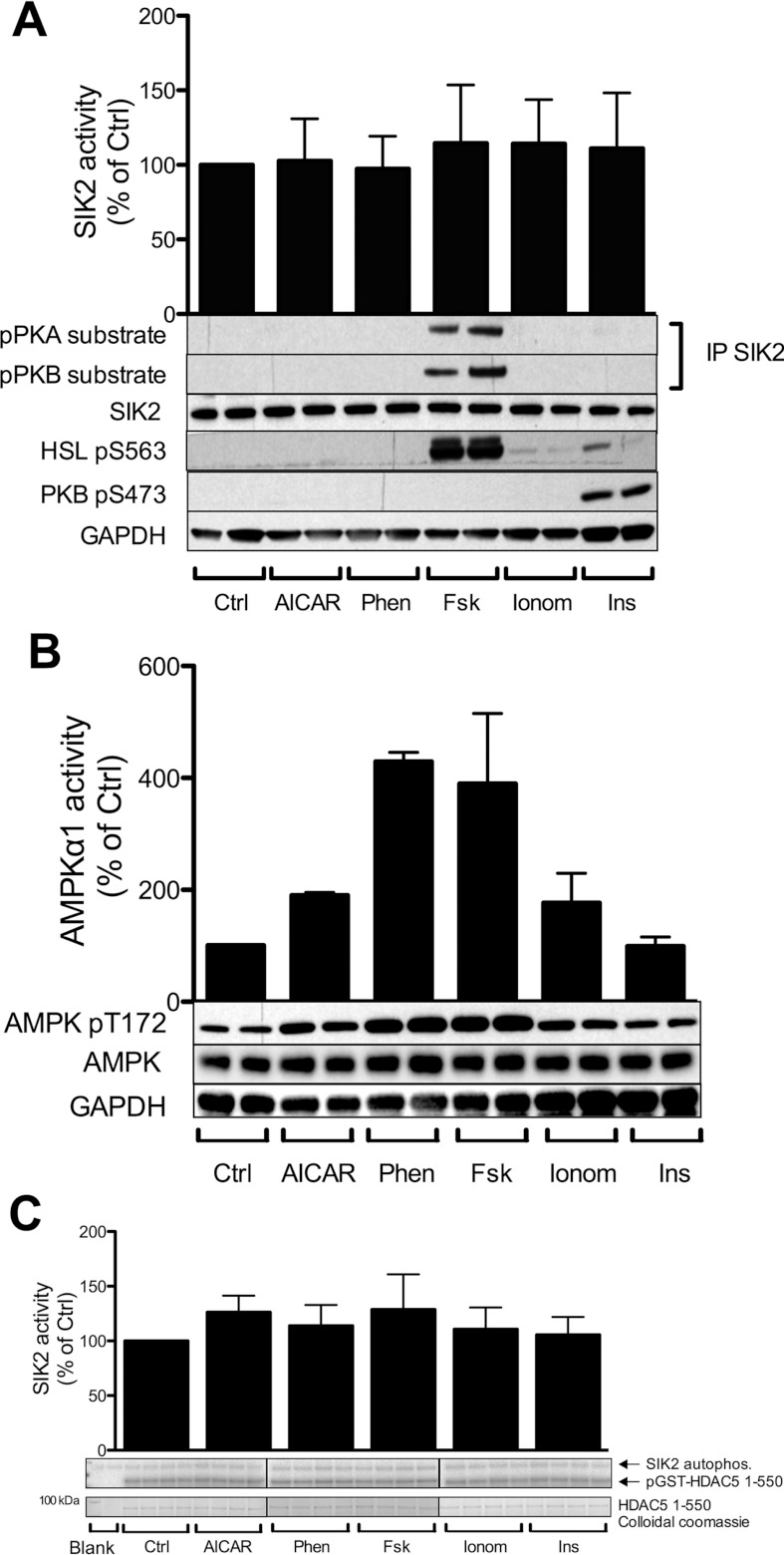
Phosphorylation and activity of SIK2 in 3T3-L1 adipocytes treated with various cellular stimuli, including cAMP inducers Fully differentiated 3T3-L1 adipocytes were treated with one of the following agents: AICAR (2 mM, 1 h), phenformin (1 mM, 1 h; Phen), forskolin (50 μM, 15 min; Fsk), ionomycin (1 μM, 5 min; Ionom) or insulin (100 nM, 10 min; Ins). (**A**) Lysates were analysed with regard to immunoprecipitated endogenous SIK2 activity *in vitro* towards the peptide substrate HDAC5tide and phosphorylation by PKA and PKB employing consensus motif (pPKA and pPKB substrate) antibodies. PKB phosphoSer^473^ and HSL phosphoSer^563^ were used as controls. Activity data presented are means±S.D. from four to six individual experiments. Blots shown are representative of 5–14 experiments. (**B**) Lysates were analysed with regard to immunoprecipitated endogenous AMPKα1 activity *in vitro* towards the peptide substrate AMARA, and phosphorylation of the T-loop using anti-phosphoThr^172^ antibodies. (**C**) Lysates were analysed with regard to immunoprecipitated SIK2 activity *in vitro* towards HDAC5 (1–550) purified protein by autoradiography as described in the Experimental section. Activity data are presented as means±S.D. from three individual experiments. Ctrl, control.

**Figure 3:**
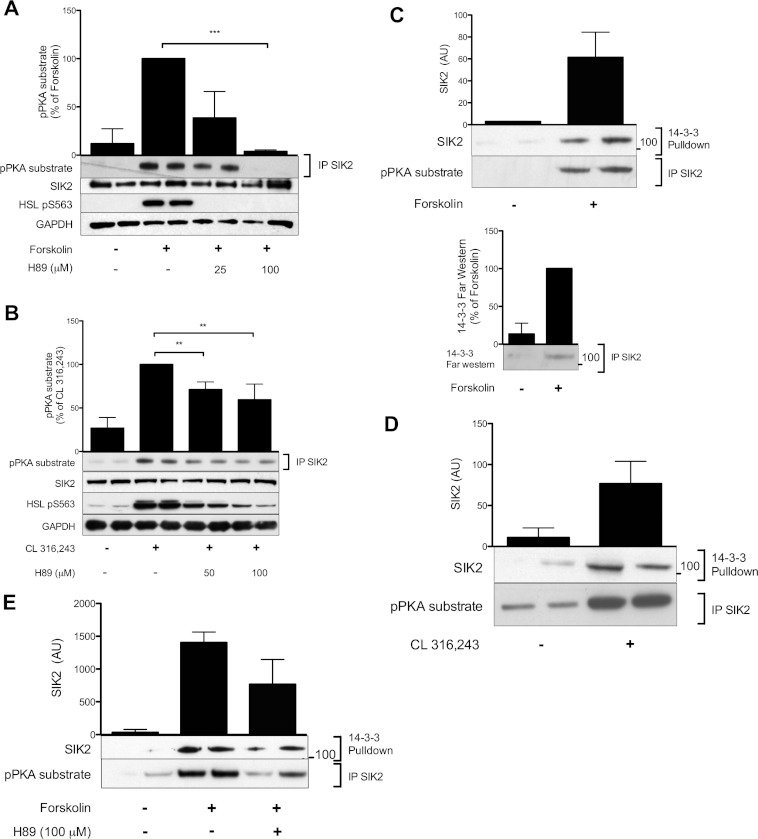
cAMP-induced phosphorylation of SIK2 coincides with 14-3-3 binding: requirement of PKA (**A** and **B**) The requirement of PKA for the phosphorylation of SIK2 was investigated in 3T3-L1 (**A**) and primary rat (**B**) adipocytes. Cells were pre-incubated for 20 min with different doses of the PKA inhibitor H89, followed by stimulation with forskolin (50 μM, 15 min) or CL 316,243 (100 nM, 30 min). Lysates were analysed with regard to total SIK2 and phosphorylation by PKA (pPKA substrate antibodies). HSL phosphoSer^563^ was used as a control. Quantified phosphorylation data are presented as means±S.D. for three to five individual experiments. **P < 0.01 and ***P < 0.001. 3T3-L1 (**C**) or rat (**D**) adipocytes were stimulated with forskolin (50 μM, 15 min) or CL 316,243 (100 nM, 30 min). 14-3-3 binding to SIK2 was demonstrated by the presence of SIK2 in 14-3-3 pull downs using GST–14-3-3ζ coupled to glutathione–Sepharose (**C**, upper panel and **D**), or by the direct interaction of recombinant yeast 14-3-3 (BMH1/BMH2) to immunoprecipitated SIK2 on a far-Western blot (**C**, lower panel). (**E**) Primary rat adipocytes were pre-incubated for 20 min with the PKA inhibitor H89, followed by stimulation with forskolin (50 μM, 15 min). The binding of 14-3-3 to SIK2 was demonstrated by the presence of SIK2 in 14-3-3 pull downs. Quantified 14-3-3-binding data from pull downs and far-Western blots are presented as means±S.D. for duplicate or single samples and are representative for two individual experiments. The blots shown are representative.

In order to investigate the role of different cellular stimuli in the regulation of SIK2, 3T3-L1 adipocytes were treated with a panel of agents, including AICAR and phenformin, known AMPK activators that affect the AMP (or ZMP)/ATP ratio, the cAMP-elevating agent forskolin, the Ca^2+^ ionophore ionomycin and insulin (Figure 2A). These stimuli were selected on the basis of their suggested role in the regulation of SIK2, AMPK or adipocyte function. The cellular stimuli were validated by monitoring the phosphorylation of target proteins. AMPK was phosphorylated on its T-loop Thr^172^ site and activated in response to AICAR, phenformin, forskolin and ionomycin (Figure 2B). PKB Ser^473^ phosphorylation was used as a positive control for activation of the insulin signalling pathway and HSL Ser^563^ phosphorylation for increased cAMP levels and the activation of PKA (Figure 2A). None of the treatments affected total SIK2 levels. The kinase activity of immunoprecipitated SIK2 was measured *in vitro* towards the peptide substrates HDAC5tide (Figure 2A) and Sakamototide (results not shown), as well as HDAC5 (1–550) recombinant protein (Figure 2C). Activity measurements did not show any significant change in response to the different treatments compared with the basal activity. However, when monitoring SIK2 phosphorylation, employing phosphoPKA- and phosphoPKB-substrate antibodies, which recognize consensus sites for PKA and PKB respectively, we detected a phosphorylation of SIK2 in response to forskolin with both antibodies (Figure 2A). Phosphorylation of SIK2 was also observed in primary rat adipocytes in response to the β_3_-adrenergic receptor agonist CL 316,243 (Figure 3B). None of the other treatments induced phosphorylation of SIK2.

To investigate the requirement of PKA for cAMP-induced phosphorylation of SIK2, we used the PKA inhibitor H89. As shown in Figures 3(A) and 3(B), pretreatment with increasing concentrations of H89 completely, or in the case of rat adipocytes partially, prevented the cAMP-induced phosphorylation of SIK2 on PKA consensus sites. To evaluate a potential role of PKB and/or the MAPK (mitogen-activated protein kinase) signalling pathway, we used a PKB inhibitor (Akti1/2) [16,23], a MEK1 [MAPK/ERK (extracellular-signal-regulated kinase) kinase 1] inhibitor (PD0325901), as well as a MAPK activator (PMA). Neither inhibition of PKB, nor activation or inhibition of the MAPK pathway (as confirmed by phosphoPKB and phosphoERK1/2 blotting) affected basal or cAMP-induced phosphorylation of SIK2 (results not shown). These results confirm an important role for PKA in the cAMP-induced phosphorylation of SIK2, although other cAMP-activated pathways, such as those regulated by Epacs (exchange protein directly activated by cAMPs), may also play a role in rat adipocytes.

### The adaptor protein 14-3-3 binds SIK2 in response to cAMP elevation in adipocytes

The T-loops of SIK1 and SIK3 have previously been shown to bind 14-3-3 proteins [9]. In the present study we demonstrated that phosphorylation of SIK2 in response to increasing levels of cAMP coincides with the binding of SIK2 to 14-3-3 proteins (Figures 3C and 3D). Endogenous SIK2 was detected in GST–14-3-3 pull downs from both 3T3-L1 adipocytes and primary rat adipocytes in response to forskolin and the β**_3_**-adrenergic receptor agonist CL 316,243 respectively (Figures 3C, upper panel, and Figure 3D). Additionally, the direct interaction between SIK2 and 14-3-3 was illustrated by the ability of recombinant 14-3-3 to bind SIK2 in a far-Western blot experiment (Figure 3C, lower panel). In the presence of H89, the binding of SIK2 to 14-3-3 proteins was decreased to the same extent as the phosphorylation (Figure 3E). This further suggests that the cAMP-induced phosphorylation of SIK2 induces its binding to 14-3-3.

### Identification of SIK2 phosphorylation sites induced by cAMP

**Figure 4:**
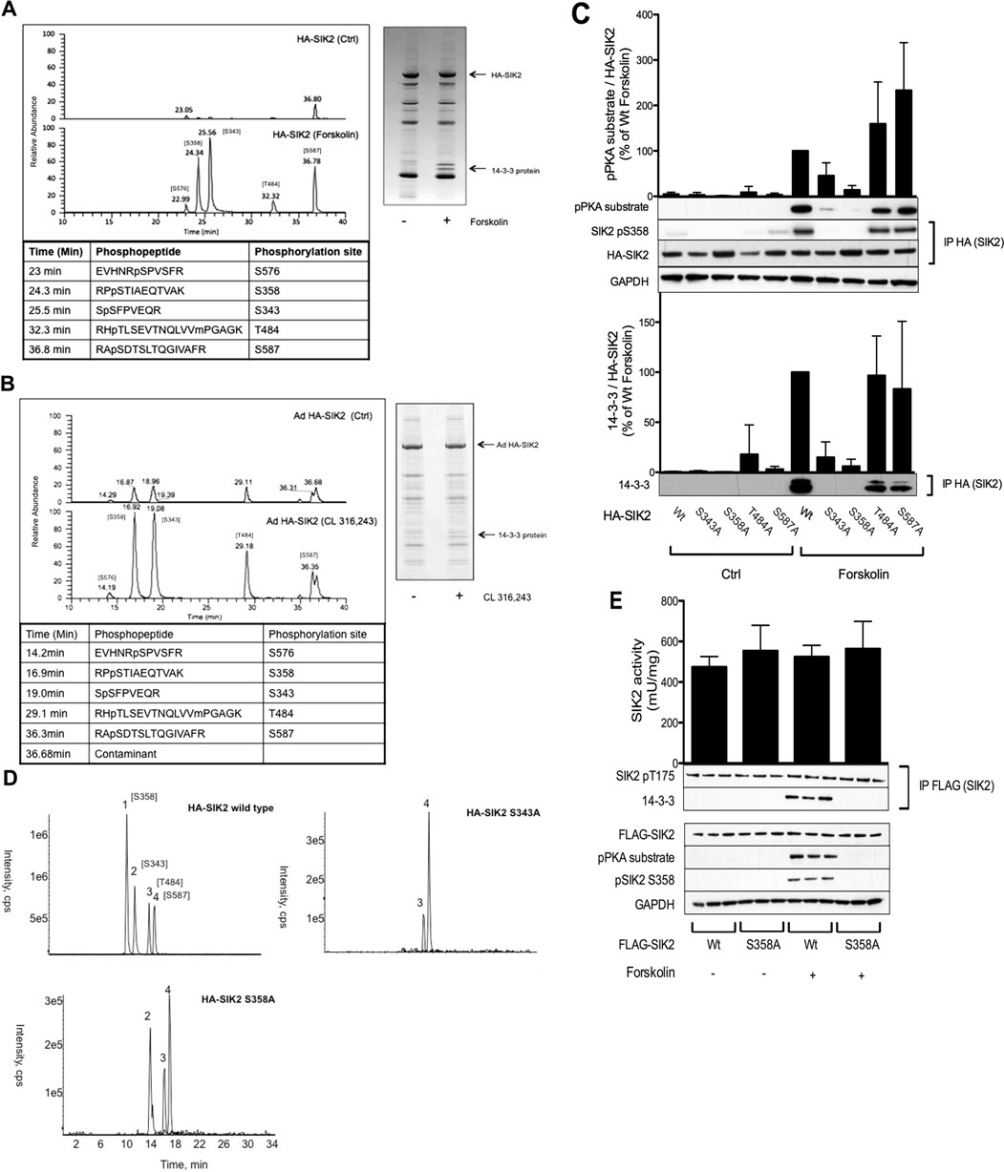
Identification and characterization of *in vivo* phosphorylation sites in SIK2 important for 14-3-3 binding (**A**) Wild-type HA–SIK2 immunopurified from forskolin-treated (100 μM, 15 min) HEK-293 cells was excised from a colloidal Coomassie Blue-stained polyacrylamide gel and subjected to trypsin digestion. Phosphopeptides were identified using LC-MS on an orbitrap classic mass spectrometer, as described in the Experimental section. Relative quantification of SIK2 phosphorylation sites is shown with the extracted ion chromatograms for the five identified phosphopeptides from HA–SIK2 isolated from control (upper panel) or forskolin treated (lower panel) HEK-293 cells. Potential 14-3-3 bands were also excised and identified by mass fingerprinting. (**B**) Primary rat adipocytes were stimulated with CL 316,243 (100 nM, 30 min) after adenoviral (Ad) expression of wild-type HA–SIK2, and SIK2 phosphopeptides and 14-3-3 isoforms were identified. Relative quantification of SIK2 phosphorylation sites is shown, but the retention times differ from those shown in (**A**), owing to the different nano-HPLC column used to separate the tryptic peptides from the HA–SIK2 isolated from adipocytes as described in the Experimental section. (**C**) Wild-type (Wt) or the indicated phosphorylation site mutant forms of SIK2 were expressed in HEK-293 cells, and their phosphorylation (upper panel) and 14-3-3 binding (lower panel) was analysed by anti-phosphoPKA substrate and anti-phosphoSer^358^ antibodies, and co-immunoprecipitation respectively. Quantified phosphorylation and 14-3-3-binding data are presented as means±S.D. for three to five independent experiments. (**D**) Wild-type HA–SIK2, Ser343Ala HA–SIK2 and Ser358Ala HA–SIK2 immunopurified from forskolin-treated (100 μM, 15 min) HEK-293 cells were excised from a colloidal Coomassie Blue-stained polyacrylamide gel and subjected to trypsin digestion. Phosphopeptides were identified as described in (**A**). Phosphorylation of Ser^358^ (1), Ser^343^ (2), Thr^484^ (3) and Ser^587^ (4) are presented as extracted ion chromatograms. (**E**) Wild-type (Wt) or Ser358Ala (S358A) FLAG–SIK2 stably expressed in HEK-293 cells were analysed with regard to T-loop (Thr^175^) and PKA (pPKA substrate) phosphorylation, 14-3-3 binding and *in vitro* kinase activity using HDAC5tide as a substrate, after treatment of the cells with forskolin (50 μM, 15 min). Activity data are presented as means±S.D. from three independent experiments. Blots shown are representative of three experiments.

In order to determine cAMP-induced phosphorylation sites in SIK2 of importance for 14-3-3 binding, a phosphopeptide mapping of SIK2 was performed (Figures 4A and 4B). Tryptic digest and analysis of phosphopeptides from wild-type HA–SIK2 expressed in HEK-293 cells revealed five sites whose phosphorylation was induced following forskolin stimulation: Ser^576^, Ser^358^, Ser^343^, Thr^484^ and Ser^587^ (Figure 4A). The same sites were detected in wild-type HA–SIK2 adenovirally expressed in primary rat adipocytes treated with CL 316,243, confirming the relevance of these sites in a more physiological setting (Figure 4B). A total of 70–90% of the protein sequence was obtained in the phosphopeptide mapping and, in addition to sites induced by cAMP elevation, we also detected peptides phosphorylated on Ser^534^, Ser^512^ (primary adipocytes) and Ser^90^ (HEK-293 cells). These phosphopeptides, however, displayed low abundancy and were therefore not studied further. The region containing potential T-loop phosphorylations, for example Thr^175^, was not covered in our analysis, probably owing to the large size of this tryptic peptide (65 amino acids). Colloidal Coomassie Blue staining, excision and mass fingerprinting of the relevant bands in the HA–IP revealed that SIK2 bound to all seven of the 14-3-3 isoforms (β, ϵ, η, γ, τ, ζ and σ) (Figures 4A and 4B).

### Ser^358^ of endogenous SIK2 is phosphorylated in primary rat adipocytes and is critical for phosphorylation and 14-3-3 binding of SIK2 in response to cAMP

Selected sites that emerged from the phosphopeptide mapping (Ser^343^, Ser^358^, Thr^484^ and Ser^587^) were subjected to site-directed mutagenesis analysis in HEK-293 cells, in order to investigate their role in the cAMP-induced phosphorylation and 14-3-3 binding of SIK2. Mutation of Thr^484^ and Ser^587^ to alanine did not affect the overall PKA phosphorylation or the 14-3-3 binding of HA–SIK2, as judged by the presence of endogenous 14-3-3 in the anti-HA–SIK2 IPs (Figure 4C). However, mutation of Ser^343^ or Ser^358^ resulted in a strong reduction of forskolin-induced phosphorylation of PKA consensus sites, and complete loss of 14-3-3 binding to SIK2 (Figure 4C). A phosphopeptide mapping of these two mutants (Figure 4D) revealed that Ser^358^ was not phosphorylated in the Ser343Ala mutant, whereas Ser^343^ was normally phosphorylated in the Ser358Ala mutant, implying that Ser^343^ is required for Ser^358^ phosphorylation and that Ser^358^ is the site directly mediating 14-3-3 binding. The results described above (Figure 2) suggest that phosphorylation and 14-3-3 binding of SIK2 does not affect its kinase activity. To further evaluate this, we examined the activity of stably expressed wild-type and Ser358Ala mutant SIK2 in HEK-293 cells. As shown in Figure 4(E), no difference in kinase activity or T-loop (Thr^175^) phosphorylation was observed between the wild-type and Ser358Ala SIK2, or following forskolin stimulation.

**Figure 5:**
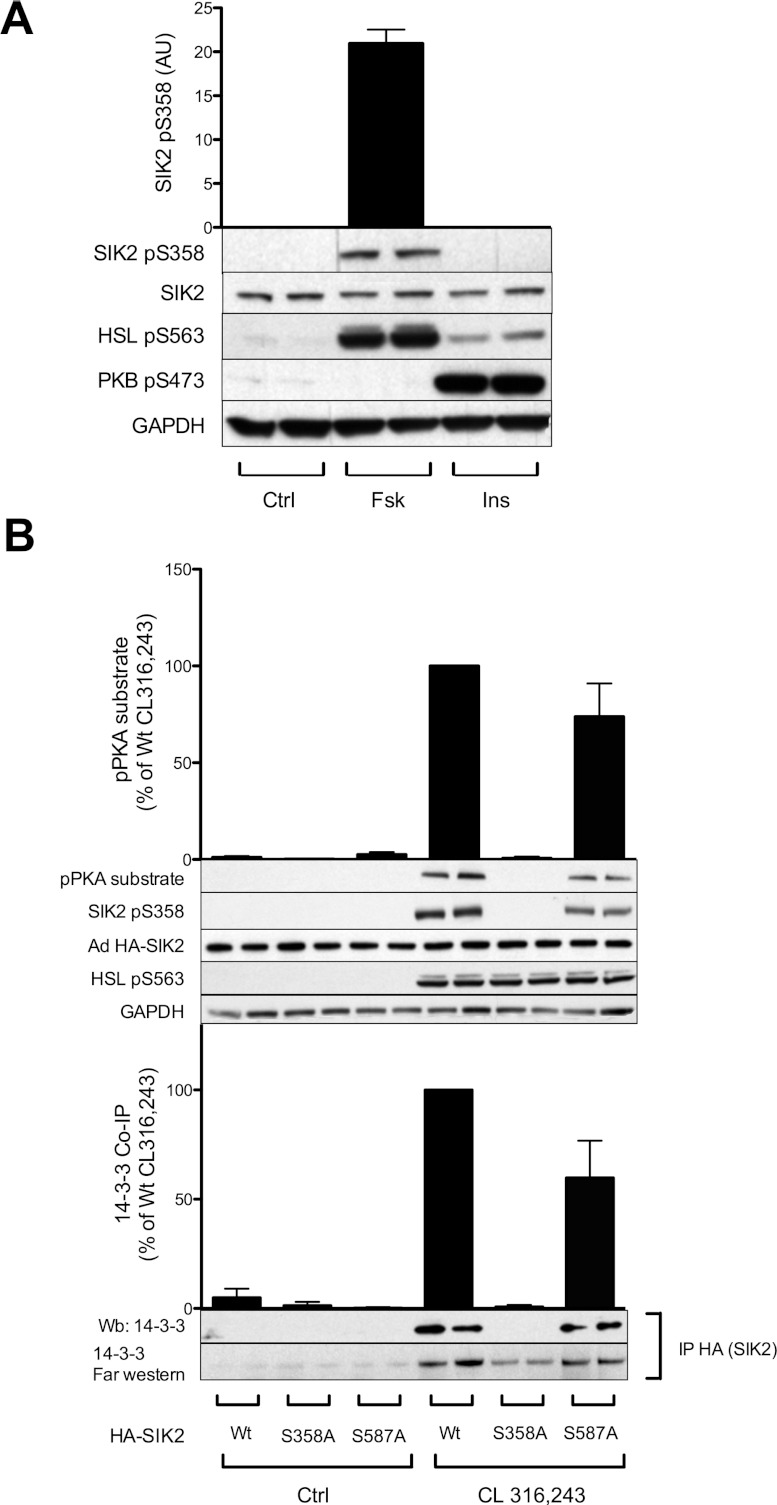
Role of Ser^358^ phosphorylation in adipocytes (**A**) 3T3-L1 adipocytes were stimulated with forskolin (50 μM, 15 min; Fsk) or insulin (100 nM, 10 min; Ins) and phosphorylation of endogenous SIK2 was analysed using a phosphospecific antibody targeting Ser^358^. Quantified phosphorylation data are presented as means±S.D. for two individual experiments. (**B**) Adenoviral vectors (Ad) encoding Ser358Ala or Ser587Ala mutant HA–SIK2 were constructed and used to evaluate the role of Ser^358^ phosphorylation in primary rat adipocytes. Wild-type (Wt), Ser358Ala (S358A) and Ser587Ala (S587A) mutant HA–SIK2 expressed in CL 316,243-treated (100 nM, 30 min) rat adipocytes were analysed with regard to phosphorylation (upper panel, pPKA substrate and phosphoSer^358^) and 14-3-3 binding (lower panel, co-immunoprecipitation and far-Western blot). Quantified data are presented as means±S.D. for two individual experiments. Blots shown are representative of two to four experiments.

To study site-specific phosphorylation of endogenous SIK2, we generated antibodies that specifically recognize SIK2 only when phosphorylated at Ser^358^ (Figure 4C). Using these antibodies we were able to demonstrate that Ser^358^ of SIK2 was indeed phosphorylated in response to forskolin in 3T3-L1 adipocytes (Figure 5A) and CL 316,243 in primary rat adipocytes (results not shown). Although PKB was activated, as measured by Ser^473^ PKB phosphorylation, no phosphorylation of Ser^358^ could be detected in response to insulin, in contrast with previous findings in the liver [11]. In addition, using adenoviral expression of wild-type, Ser358Ala and Ser587Ala HA–SIK2, Ser^358^ was confirmed as the major site responsible for the PKA-dependent phosphorylation of SIK2 in adipocytes (Figure 5B, upper panel). Ser587Ala HA–SIK2 was phosphorylated almost to the same extent as the wild-type HA–SIK2 upon stimulation with CL 316,243, suggesting that Ser^587^ is not a major site induced by cAMP in adipocytes. Furthermore, wild-type and Ser587Ala HA–SIK2, but not the Ser358Ala mutant, was demonstrated to directly bind 14-3-3 in CL 316,243-stimulated rat adipocytes, as detected using co-immunoprecipitation and far-Western blotting (Figure 5B, lower panel).

### No evidence of nucleo–cytoplasmic shuttling of SIK2 exogenously expressed in HEK-293 cells

**Figure 6:**
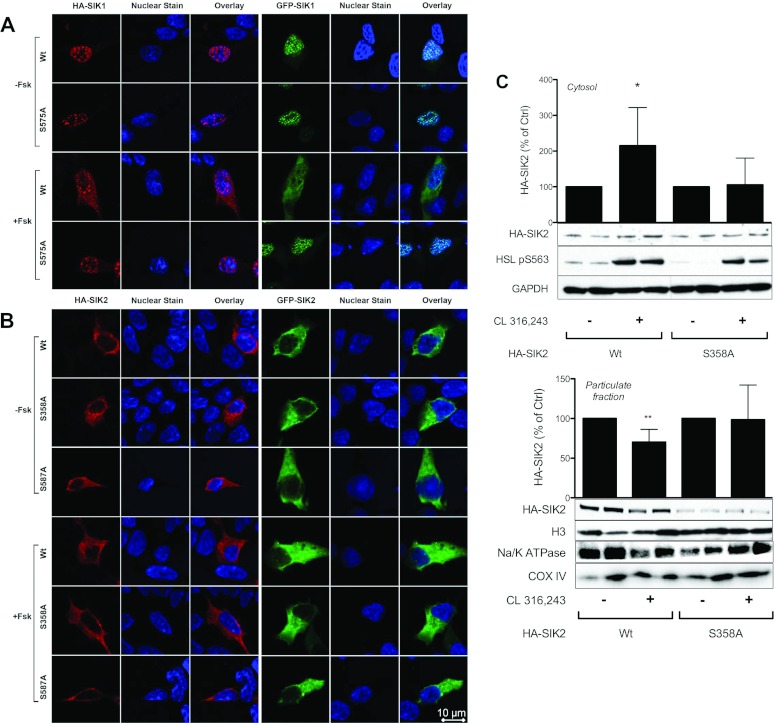
Confocal imaging of SIK1 and SIK2 in HEK-293 cells and fractionation of primary rat adipocytes after treatment with cAMP inducers HEK-293 cells were transfected with either HA- or GFP-tagged wild-type (Wt) or Ser575Ala (S575A) SIK1 (**A**) or wild-type (Wt), Ser358Ala (S358A) or Ser587Ala (S587A) SIK2 (**B**). At 16 h post-transfection, cells were stimulated with forskolin (50 μM, 30 min) and subsequently fixed and stained as described in the Experimental section. The cells shown in the Figure are representative of two independent experiments. (**C**) Wild-type (Wt) or Ser358Ala (S358A) mutant HA–SIK2 was expressed in primary rat adipocytes, which were stimulated with CL 316,243 (100 nM, 30 min). The adipocytes were then homogenized and centrifuged at 60000 ***g*** to obtain cytosol and crude particulate fractions. The fractions were identified using markers for cytosol (GAPDH), nuclei (H3), plasma membrane (Na/K ATPase) and mitochondria (COX IV). HA–SIK2 protein was analysed. Since the expression level of the Ser358Ala mutant was often lower than the wild-type, the non-stimulated sample for each construct was set to 100%. Quantified data are presented as means±S.D. for seven to nine independent experiments. Blots shown are representative. *P<0.05, **P<0.01.

HEK-293 cells were used to study the localization of HA- or GFP-tagged SIK2 in the absence or presence of the cAMP-elevating agent forskolin. The localization of wild-type and Ser575Ala (Ser^577^ in mouse and rat) SIK1 was monitored as a control. Confocal imaging revealed that the subcellular localization of wild-type and Ser575Ala SIK1 was nuclear in non-treated cells (Figure 6A). Upon stimulation with forskolin, wild-type SIK1 translocated to the cytosol, whereas Ser575Ala SIK1 remained in the nucleus (Figure 6A). This is in agreement with previous findings describing an NLS in the C-terminal region of SIK1 and a nucleo–cytoplasmic shuttling regulated by cAMP via phosphorylation of Ser^577^/Ser^575^ [7,13,24]. In contrast with SIK1, wild-type SIK2 and the mutant variants Ser358Ala and Ser587Ala were all cytoplasmic rather than nuclear in non-treated cells (Figure 6B). Furthermore, all three SIK2 variants remained cytoplasmic after forskolin treatment (Figure 6B). The results of the present study therefore suggest SIK2 to be cytoplasmic, with no obvious nucleo–cytoplasmic shuttling in response to cAMP elevation, when overexpressed in HEK-293 cells. We could not confirm earlier findings suggesting that Ser587Ala is of importance for a nuclear export of SIK2 [3]. Our findings were consistent, independent of the epitope tag used for detection of SIK1 and SIK2.

### Phosphorylation-dependent relocalization of SIK2 in response to β-adrenergic stimulation of adipocytes

A common function of 14-3-3 proteins is to trap its binding partners in the cytosol [25], and we therefore more specifically investigated the localization of exogenously expressed SIK2 in adipocytes, using a fractionation protocol. Primary rat adipocytes expressing wild-type or Ser358Ala HA–SIK2 were stimulated, homogenized and centrifuged (60000 ***g***) to obtain a cytosol and crude particulate fractions. The fractions were characterized using cytosolic (GAPDH), nucleic (H3), mitochondrial (COX IV) and plasma membrane (Na/K ATPase) markers. The particulate fraction contained nuclei, mitochondria and plasma membrane (Figure 6C), but no GAPDH (results not shown). GAPDH was the only detectable marker in the cytosol fraction. As shown in Figure 6(C), a significant increase in exogenously expressed wild-type HA–SIK2, but not the Ser358Ala mutant, was found in the cytosolic fraction upon treatment of rat adipocytes with CL 316,243 (*P*=0.0121). This coincided with a significant decrease in wild-type HA–SIK2 in the crude particulate fraction (*P*=0.0027), suggesting a relocalization of SIK2 upon cAMP stimulation. These data demonstrate a functional importance of Ser^358^ phosphorylation for the regulation of SIK2 localization in response to cAMP in adipocytes.

## DISCUSSION

The present study is the first to investigate the regulation of SIK2 in response to cAMP in adipocytes, a highly relevant cell type for SIK2 [2] and, in agreement with previous findings, we report a cAMP-induced phosphorylation of SIK2 [3,14]. An important conclusion from the present study is that, in adipocytes, Ser^358^, rather than Ser^587^, is the main site phosphorylated and responsible for the binding of SIK2 to 14-3-3 proteins. In contrast with SIK1, SIK3 and the Par-1/MARK family of AMPK-related kinases, which bind 14-3-3 in resting cells [9,26], binding of SIK2 to 14-3-3 proteins is dynamic, taking place only after stimulation of the cAMP/PKA pathway, re-enforcing a physiological role for this interaction.

In previous studies performed in Y1 cells and 3T3-L1 fibroblasts, Ser^577^ of SIK1, and the corresponding site in SIK2 Ser^587^, was suggested to be the phosphorylation site targeted by cAMP regulation [3,14]. These conclusions were drawn on the basis of sequence motif analysis and site-directed mutagenesis, and it is therefore not clear whether other sites are also phosphorylated in these systems. We used a more direct strategy and identified all cAMP-induced SIK2 phosphorylation sites in both HEK-293 cells and adipocytes. Although we found that Ser^587^ of SIK2 is indeed phosphorylated in response to cAMP in adipocytes, mutation of this residue only resulted in a moderate reduction in total PKA phosphorylation, as measured by consensus blotting of PKA phosphorylation sites on SIK2, and did not affect the 14-3-3 binding of SIK2. Mutation of Ser^358^ on the other hand, resulted in a complete loss of cAMP-induced phosphorylation and 14-3-3 binding in both HEK-293 cells and adipocytes. We therefore conclude that Ser^358^ is the main site phosphorylated in response to cAMP, and clearly the most important one for the binding of SIK2 to 14-3-3. We also show that this site is phosphorylated at an endogenous level. Another cAMP-induced site identified was Ser^343^, the mutant of which was also not able to bind 14-3-3. When analysing phosphopeptides in the Ser358Ala and Ser343Ala mutants, we noted that Ser^358^ was not phosphorylated in the Ser343Ala mutant, a finding that was confirmed using anti-phosphoSer^358^ antibodies. This suggests that Ser^343^ may be required for Ser^358^ phosphorylation to occur. As Ser^343^ was normally phosphorylated in the Ser358Ala mutant, which was unable to bind 14-3-3, we propose that Ser^358^, and not Ser^343^, is the site with which 14-3-3 interacts. With an arginine residue positioned at −5 and −3 from the phosphorylated serine, Ser^358^ is situated in a sequence that is one of the most common motifs for 14-3-3 binding in mammalian proteins [27].

The effect of cAMP-induced phosphorylation on the intrinsic kinase activity of SIK isoforms was previously poorly investigated. We show that neither cAMP elevation or mutation of PKA-targeted sites affect the *in vitro* kinase activity of SIK2 towards peptide or protein substrates, implicating another role for the phosphorylation we observed. A reported consequence of phosphorylation-induced binding of proteins to 14-3-3 is a change in their subcellular localization, commonly an accumulation in the cytosol [25]. Ser^577^ phosphorylation of exogenously expressed SIK1 has been shown to result in its export from the nucleus of Y1 cells and 3T3-L1 fibroblasts in response to cAMP elevation, a result that was reproduced by us in HEK-293 cells [3,7,14]. In previous studies of SIK2 in 3T3-L1 fibroblasts, the wild-type protein was, in contrast with SIK1, virtually absent from the nucleus of resting cells, and did not change in response to cAMP [3], similar to our results in HEK-293 cells. However, on the basis of an increased presence of the Ser587Ala SIK2 mutant in the nucleus, compared with the wild-type, as well as the ability of SIK2 to repress gene expression from a Cre reporter, it was concluded by previous investigators that SIK2 is most likely also subject to cAMP-induced nucleo–cytoplasmic shuttling [3]. In our HEK-293 cell experiments, using confocal microscopy and two differently tagged versions of the kinases, we found no evidence of a difference in localization between wild-type, Ser587Ala or Ser358Ala SIK2, or following forskolin treatment. On the basis of the present and previous studies, the subcellular localization and its potential regulation by cAMP, appears to be distinctly different for SIK1 and SIK2, at least when these kinases are exogenously expressed in 3T3-L1 fibroblasts or HEK-293 cells. This is probably due to the presence of an NLS, located close to Ser^577^ in SIK1, whereas no such sequence is apparent in SIK2 [14]. Exactly how phosphorylation of SIK1 induces its relocalization to the cytoplasm has not been determined, but 14-3-3 binding to Ser^577^, thereby masking the NLS, could be a potential mechanism, a possibility that to our knowledge has not been investigated.

Adipocytes represent a more physiologically relevant model for studies of SIK2 than HEK-293 cells, and we therefore also wanted to investigate potential regulation of SIK2 localization in these cells. Primary adipocytes are not suitable for use in immunocytochemistry experiments, due to their large lipid droplet and thin cytoplasm, and we therefore instead used a fractionation approach. Since our studies in HEK-293 cells did not support a relocalization of SIK2 from the nucleus, we prepared a crude particulate fraction in order to also monitor non-nuclear membranes. In these experiments, we detected a significant increase in the wild-type HA–SIK2 protein in the cytosol following cAMP elevation, and this was paralleled by a reduced amount of SIK2 in the particulate fraction. This relocalization was mediated by phosphorylation of Ser^358^, as the PKA-insensitive Ser358Ala mutant did not display any change in localization. From the present study we cannot point out which membrane compartment, nuclear or extra-nuclear, that SIK2 relocates from in adipocytes. Nevertheless, we hypothesize that phosphorylation-induced relocalization of SIK2, mediated by 14-3-3 binding, is important for its cellular function, possibly restricting its access to downstream substrates.

In agreement with previous studies in which SIK2 activity was not significantly affected by AICAR or phenformin, in mouse embryonic fibroblasts, HeLa cells [8] or in skeletal muscle [10], the present study does not support an activation of SIK2 in response to changes in cellular AMP/ATP levels in 3T3-L1 adipocytes. AMPK activation upon AICAR treatment or other agents that increase the AMP/ATP ratio [28] requires the binding of AMP to the γ-subunit of AMPK [29,30]. AMP binding or binding to any protein with the characteristics of a γ-subunit has not yet been demonstrated for SIK2 or any of the other AMPK-related kinases [9].

In the liver, insulin has previously been proposed to induce an activation of SIK2 via PKB phosphorylation of Ser^358^ [11]. This regulation was also studied in HEK-293 cells, in which a phosphorylation site mutant (Ser358Ala) was shown not to be phosphorylated. Another study reported SIK2 to be phosphorylated at Ser^587^, rather than Ser^358^, upon insulin stimulation of brown adipocytes [31]. In the present study using 3T3-L1 adipocytes, although PKB was clearly activated, we did not observe an insulin-induced phosphorylation of endogenous SIK2. Our results do not support a role for insulin in the regulation of Ser^358^ or Ser^587^ phosphorylation of SIK2 in adipocytes, but rather suggest that these sites are induced by cAMP. The fact that PKB was not activated in response to forskolin suggests that the SIK2 signal observed with an antibody recognizing PKB consensus motifs following forskolin treatment is due to the overlap between PKA and PKB consensus sequences. We also used a PKB inhibitor, as well as a MAPK activator and inhibitor, to rule out that these signalling pathways were involved in the forskolin-induced phosphorylation observed with the phosphoPKB and phosphoPKA substrate antibodies (results not shown).

AMPK is known to be activated in response to elevation of intracellular [Ca^2+^] in 3T3-L1 adipocytes [32] and other cells [33–35]. Furthermore, a number of reports propose that AMPK-related kinases, including Thr^484^ of SIK2 [12], may be regulated by Ca^2+^ signalling [33–37]. Although AMPK phosphorylation and activation was observed in our experiments, treatment with a Ca^2+^ ionophore had no effect on SIK2 activity or its phosphorylation by PKA or PKB, suggesting that Ca^2+^-regulated signalling components [e.g. CaMK or CaMKK (CaMK kinase)] do not participate in SIK2 regulation in adipocytes.

A key question that remains to be addressed is the biological function of SIK2 in adipocytes, and how it is affected by the regulation we describe. Recently, a total body SIK2 knockout mouse model was described, with no major effects on body mass [38]. One could speculate that other SIK isoforms are up-regulated as a compensatory mechanism in these mice, and a more precise examination of the adipose tissue is needed to reveal the role of SIK2 in adipose tissue. In other cell types, including β-cells, melanocytes and hepatocytes, SIKs are suggested to repress gene expression by phosphorylating, and thereby reducing the nuclear presence of, transcriptional regulators, like CRTCs [11,15,38,39]. cAMP/PKA-mediated phosphorylation has been suggested to inhibit SIK2 function, leading to CRTC activation. However, no precise mechanism underlying this inhibition was presented in these studies. In the present study we have shown that, in adipocytes, cAMP signals may restrict, or potentially stimulate, SIK2 action, not by affecting its intrinsic kinase activity, but rather by inducing its phosphorylation of Ser^358^, binding to 14-3-3 and a subsequent movement away from potential substrates. We hypothesize that SIK2 in this manner could mediate effects of cAMP on adipocyte gene expression or participate in the acute regulation of lipid storage by cAMP, such as catecholamine-induced lipolysis.
